# Prefrontal Cortex Activation During Verbal Fluency Task and Tower of London Task in Schizophrenia and Major Depressive Disorder

**DOI:** 10.3389/fpsyt.2021.709875

**Published:** 2021-10-08

**Authors:** Yilei Xiang, Yuan Li, Chang Shu, Zhongchun Liu, Huiling Wang, Gaohua Wang

**Affiliations:** ^1^Department of Psychiatry, Renmin Hospital of Wuhan University, Wuhan, China; ^2^Hubei Provincial Key Laboratory of Developmentally Originated Disease, Wuhan University, Wuhan, China; ^3^Department of Psychiatry, Zhongxiang Hospital of Renmin Hospital of Wuhan University, Zhongxiang, China; ^4^Hubei Institute of Neurology and Psychiatry Research, Renmin Hospital of Wuhan University, Wuhan, China

**Keywords:** SCH, MDD, functional NIRS, VFT, TOL

## Abstract

**Background:** Cognitive dysfunction is a common clinical feature of mental disorders. A number of functional near-infrared spectroscopy (fNIRS) studies have shown reduced prefrontal activation during the verbal fluency task (VFT) in schizophrenia (SZ) and major depressive disorder (MDD). However, no studies have examined and compared the brain activation patterns during the Tower of London (TOL), which is another classic, high-sensitivity executive function testing tool, in these two serious mental disorders. This study aimed to assess the characteristics of brain activation during the two different cognitive tasks in SZ and MDD patients.

**Methods:** This study recruited 30 patients with SZ, 30 patients with MDD, and 30 demographically matched healthy controls (HCs). The hemodynamic changes of the prefrontal cortex (PFC) were measured using 32-channel fNIRS during performance of the TOL task and VFT task.

**Results:** SZ patients showed poorer VFT performance than MDD patients and HCs, and the two patient groups showed poorer TOL performance than HCs. Compared to HCs, both of the patient groups exhibited a significant decreased activation in the extensive PFC. Particularly in certain channels in the dorsolateral PFC (DLPFC), SZ patients exhibited significantly decreased hemodynamic changes than the MDD patients.

**Conclusions:** Patients with SZ and MDD have different levels of impairment in different cognitive domains and different patterns of brain activation during the two cognitive tasks. Further research is needed to determine the use of fNIRS for clinical evaluation and diagnosis.

## Introduction

Cognitive dysfunction is gaining attention as a common clinical feature of mental disorders, which has a large impact on quality of life and long-term prognosis. Cognitive dysfunction is considered to be a core feature of schizophrenia (SZ) in addition to other positive and negative symptoms (1). Cognitive function starts to decline many years before the first psychotic symptoms manifest themselves in the context of SZ (2). Major depressive disorder (MDD) is characterized by impaired affect, cognitive dysfunction, and significant psychosocial impairment that may persist from weeks to years. It suggested that the cognitive dysfunction in MDD persists following symptomatic remission, which may contribute to social dysfunction and suicide ideation (3).

Many neuroimaging studies have demonstrated that cognitive deficits in patients with SZ and MDD are associated with prefrontal cortex (PFC) dysfunction (4, 5). A fMRI study provided evidence that salience network abnormality may play a critical role in the pathogenesis of these two mental disorders (6). Multichannel functional near-infrared spectroscopy (fNIRS) is a relatively new method for investigating the hemodynamic activity of the cerebral cortex. Compared with other neuroimaging methodologies (such as fMRI or SPECT), fNIRS has superior time resolution and can be used flexibly. Unlike EEG and MEG, its data are not much susceptible to electrical noise, since it is an optical imaging modality (7).Because of its wide applicability, more and more researchers are using fNIRS to study brain function in psychiatric disorders (8).

The verbal fluency task (VFT) is a representative cognitive task in the fNIRS studies to assess executive function, which is regarded as associated with the function of PFC. The classic VFT takes two forms, phonemic or semantic word fluency, requiring participants to generate as many words as possible beginning with a certain letter or belonging to a certain category of words (9). Many studies have found hypofunction of PFC in SZ or MDD during VFT (10, 11). However, VFT only covers a restricted aspect of executive function. Different areas of tasks are needed to explore the cognitive function in patients with mental disorders.

The Tower of London (TOL) task is another classic, high-sensitivity executive function testing tool that mainly reflects planning and problem-solving abilities (12). The TOL task requires participants to apply many types of ability, such as complex visual and spatial planning, working memory, and selective attention (13). A previous fNIRS study found reduced prefrontal activation during TOL in first-episode SZ (14). A study compared cognitive and executive function in SZ and MDD patients, which showed that patients' performance was lower than HCs, and SZ performed worse than MDD (15). However, no study has compared the brain activation patterns between SZ and MDD patients during the TOL task and VFT task using fNIRS. Taken together, this study aimed to assess the different characteristics of brain activation in SZ and MDD patients during the VFT and TOL task. Furthermore, we are interested in finding whether fNIRS can distinguish between these two mental disorders.

## Methods

### Participants

This study recruited 30 patients with SZ and 30 patients with MDD from the Psychiatry Department of Renmin Hospital of Wuhan University in China, from December 2015 to August 2016. Thirty demographically matched healthy volunteers were taken to serve as healthy controls (HCs). The patients were diagnosed by two experienced psychiatrists according to the DSM-V criteria. All participants were aged from 18 to 50 years. They had normal IQ and received education in junior high school and above. Exclusion criteria included neurological or severe somatic disease, alcohol/substance abuse, and uncooperative patient. The Positive and Negative Syndrome Scale (PANSS) was used to evaluate psychiatric symptoms in patients with SZ (16). The depressive and anxious symptoms were evaluated by the Hamilton Rating Scale for Depression (HAMD) and Hamilton Rating Scale for Anxiety (HAMA) in MDD patients (17, 18). All of the patients enrolled were taking medications, and the medication information is shown in Table 1. Daily doses of all antipsychotics were converted to an equivalent dose of chlorpromazine; antidepressants, to that of imipramine; and anxiolytics, to that of diazepam (19). The ethics committees of Renmin Hospital of Wuhan University approved the study. Written informed consent was obtained from all subjects.

**Table 1:** Demographics, clinical information, and task performance.

	**SZ (*n* = 30)**	**MDD (*n* = 30)**	**HC (*n* = 30)**	***P***
Age (years)	27.23 ± 7.04	29.40 ± 8.87	27.27 ± 7.90	0.483
Gender (men/women)	14/16	12/18	12/18	0.835
Education (years)	13.90 ± 2.44	14.33 ± 2.45	15.20 ± 1.65	0.086
Duration of illness (months)	28.71 ± 31.14	26.71 ± 24.72	–	–
PANSS	72.1 ± 6.58	–	–	–
HAMD	–	25.2 ± 6.65	–	–
HAMA	–	17.5 ± 6.77	–	–
Medicine (mg/day)				
Antipsychotics	412.50 ± 181.20^A^	150.00 ± 32.07^A^	–	–
Antidepressants	41.90 ± 17.71^B^	86.67 ± 7.93^B^	–	–
Anxiolytics	5.30 ± 2.60^C^	6.18 ± 0.53^C^	–	–
VTF performance (*n*)	7.65 ± 2.33^a,b^	9.53 ± 2.45	9.76 ± 1.83	0.001^*^
TOL performance				
Total responses (*n*)	11.87 ± 5.34^b^	12.43 ± 5.08^c^	17.63 ± 3.75	0.000^*^
Correct responses (*n*)	7.80 ± 5.25^b^	9.40 ± 4.70^c^	14.03 ± 4.93	0.000^*^
Accuracy (%)	0.64 ± 0.22^a,b^	0.75 ± 0.24	0.79 ± 0.22	0.020^*^
Average responses time (*s*)	12.39 ± 7.43^b^	10.57 ± 2.94^c^	8.45 ± 2.18	0.008^*^

*PANSS, Positive and Negative Syndrome Scale; HAMD, Hamilton Depression Scale; HAMA, Hamilton Anxiety Rating Scale; SZ, Schizophrenia; MDD, Major depressive disorder; HC, Healthy control*.

a *The difference between SZ and HC is significant*.

b *The difference between SZ and MDD is significant*.

c *The difference between MDD and HC is significant*.

A *The chlorpromazine equivalent dose*.

B *The imipramine equivalent dose*.

C *The diazepam equivalent dose*.

* *means that the difference is statistically significant*.

### Activation Task

This study used the VFT and the TOL as cognitive tasks. Participants were asked to complete both tasks on the same day. The VFT was designed in four blocks consisting of a 30-s task period and a 30-s rest period, which was similar to previous studies (12, 20). At the beginning of the task, there was a 30-s pre-scanning. The participants needed to list as many items as possible that belong to the specific category (four-legged, vegetables, household appliances, and fruits) during the task period, while repeat counting from 1 to 5 in the rest period. The task performance was the mean number of the correct items of the four blocks. The TOL had six blocks, which also consist of a 30-s task period and a 30-s rest period. During the task period, the participants were asked to observe two pictures (A and B) on the computer screen, image how many moves will have to be made to make the arrangement of balls in picture “A” look like the arrangement of balls in picture “B,” and pressed the key that corresponds with the number of moves. Then, the computer switched to the next pictures automatically. The subjects would do nothing but sit during the rest period. The accuracy and average response time were recorded as the task performance.

### fNIRS Measurements

This study used a 32-channel fNIRS machine (CW5, TechEn Inc., American) to measure the relative concentration changes of oxygenated hemoglobin ([oxy-Hb]) and deoxyhemoglobin ([deoxy-Hb]) using two wavelengths (695 and 830 nm) of infrared light, based on the modified Beer–Lambert law. In this system, 3^*^7 probe arrangement was adopted, consisting of 11 light sources and 10 light detectors. The distance between pairs of probes was set at 3.0 cm, and the temporal resolution was set to 0.02 s. The measurement area between a detector and source probe pair was defined as a channel (ch). The source–detector probes were placed on the prefrontal areas. According to the international 10–20 system, the 32 channels' positions were as follows: ch3, ch7, ch11–14, ch19–22, ch24, ch26–27, ch29–30, and ch32 in dorsolateral PFC (DLPFC); ch1–2, ch9–10, ch23, and ch31 in ventrolateral PFC (VLPFC); and ch4–6, ch8, ch15–18, ch25, and ch28 in frontopolar PFC (FPPFC) based on Brodmann's area (21) (see **Figure 3**).

### fNIRS Pre-processing

This study used HOMER, a MATLAB-based graphical user interface program to analyze the functional brain data from NIRS (22). First, in order to remove instrument noise and physiological interference, the raw data were filtered using bandpass filtering techniques within the range of 0.01–0.2 Hz. Then, the optical density was translated to hemoglobin concentrations. We focused on [oxy-Hb] because the change of [oxy-Hb] is a more direct response to task-related brain activation (23). The last 5 s of the rest period was used as the baseline. We calculated the average [oxy-Hb] of the task period and baseline in each channel for each participant during the VFT and TOL task. Then, the mean [oxy-Hb] change was calculated by subtracting the baseline mean values from the task period mean values.

### Statistical Analysis

All data were imported into SPSS (version 26.0) for statistical analyses. One-way analysis of variance (ANOVA) was used to compare differences in demographic characteristic and task performance among HC, SZ, and MDD; Kruskal-Wallis test was used when variance between groups was not homogeneous. Sex-based group difference was evaluated using the chi-square test. Paired *t*-test was performed to compare the average [oxy-Hb] of each channel during the task period and baseline to determine which channels were activated during tasks. Then, two-way ANOVA was performed to compare the mean [oxy-Hb] change of each channel during different cognitive tasks between groups. *Post-hoc* comparisons were made with the LSD method to reveal the source of ANOVA. The false discovery rate (FDR) was utilized to correct for multiple comparisons. Significance level was set at a *P* < 0.05.

## Results

### Demographics, Clinical Information, and Task Performance

There were no significant differences in age, gender, and education between the two patient groups and HCs. The SZ patients showed poorer VFT performance than the MDD patients and HCs, and the mean number of the correct items there was significantly lower. The two patient groups showed poorer TOL performance than HCs. The accuracy of SZ patients was statistically lower than HCs, and the average response time of SZ patients was longer. MDD patients also had longer average responses time than HCs, but there was no difference in the accuracy. However, the accuracy of MDD patients was statistically higher than SZ patients (see Table 1).

### PFC Activation During the Task

**Figure 1:**
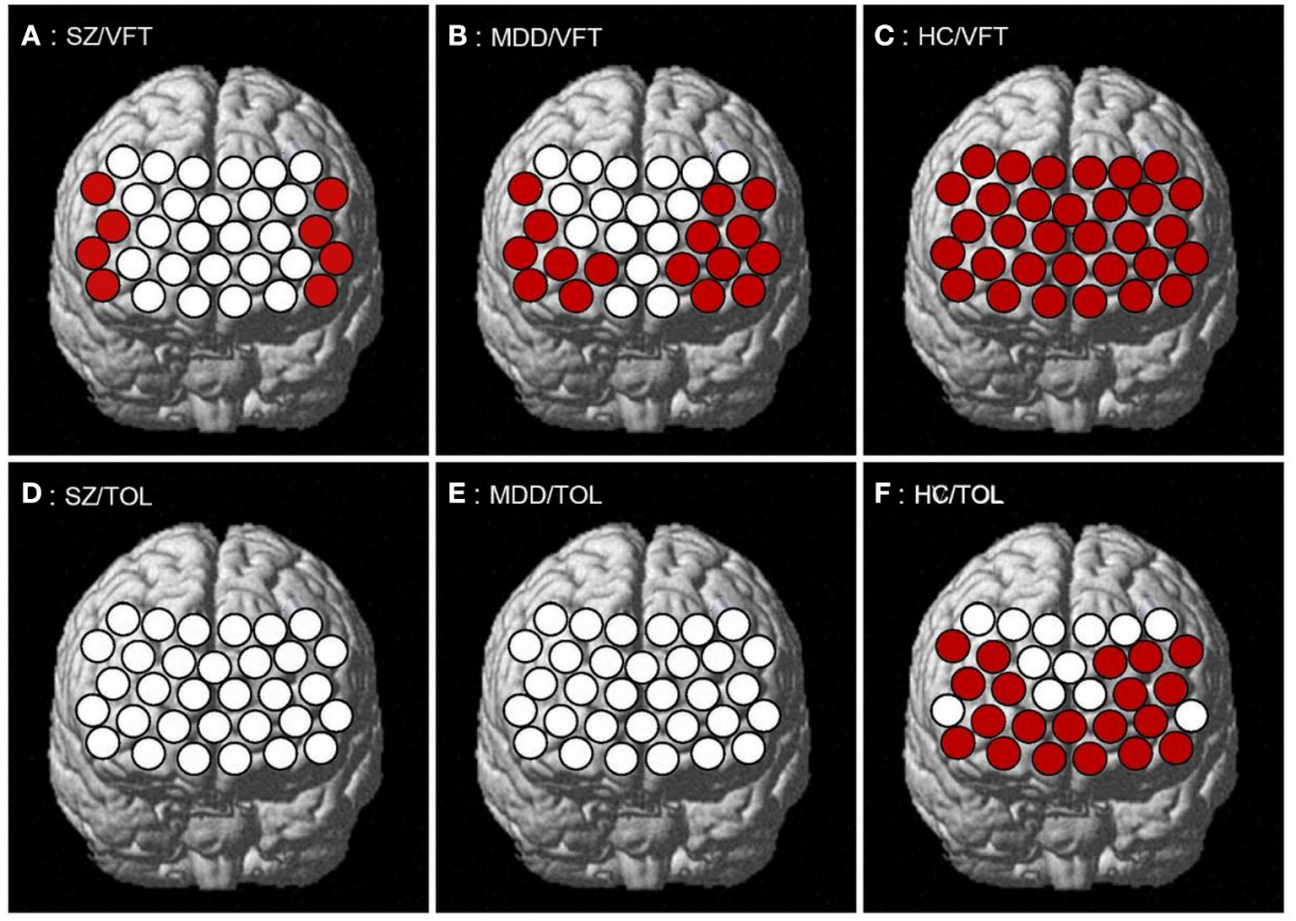
PFC activation during the task. **(A–C)** respectively represents the PFC activation of SZ, MDD, HC in VFT; **(D–F)** respectively represents the PFC activation of SZ, MDD, HC in TOL.

SZ patients showed a significant increase in the eight channels of the PFC (ch1–2, ch9–10, ch12, ch21, ch23, and ch31 in the VLPFC and part of the DLPFC; *t* = 2.473–5.180, FDR *p* = 0.019–0.049) during the VTF task, while none of the 32 channels had significant activation in the TOL task. MDD patients showed a significant increase in 16 channels of the PFC (ch1–3, ch5, ch7–12, ch19–23, and ch31 in the VLPFC, DLPFC, and part of the FPPFC; *t* = 2.108–4.525, FDR *p* = 0.011–0.044) during the VFT task; also, there was no significant activation in all channels during the TOL task. HCs showed a significant increase in all channels (*t* = 2.183–9.112, FDR *p* = 0.013–0.047) of the PFC during the VFT task and in 20 channels (ch1, ch3–9, ch11–15, ch19–23, ch28, and ch31 in the VLPFC, DLPFC, and FPPFC; *t* = 2.064–4.901, FDR *p* = 0.021–0.048) during the TOL task (see Figure 1).

### Group Comparison of the Degree of the PFC Activation

**Figure 2:**
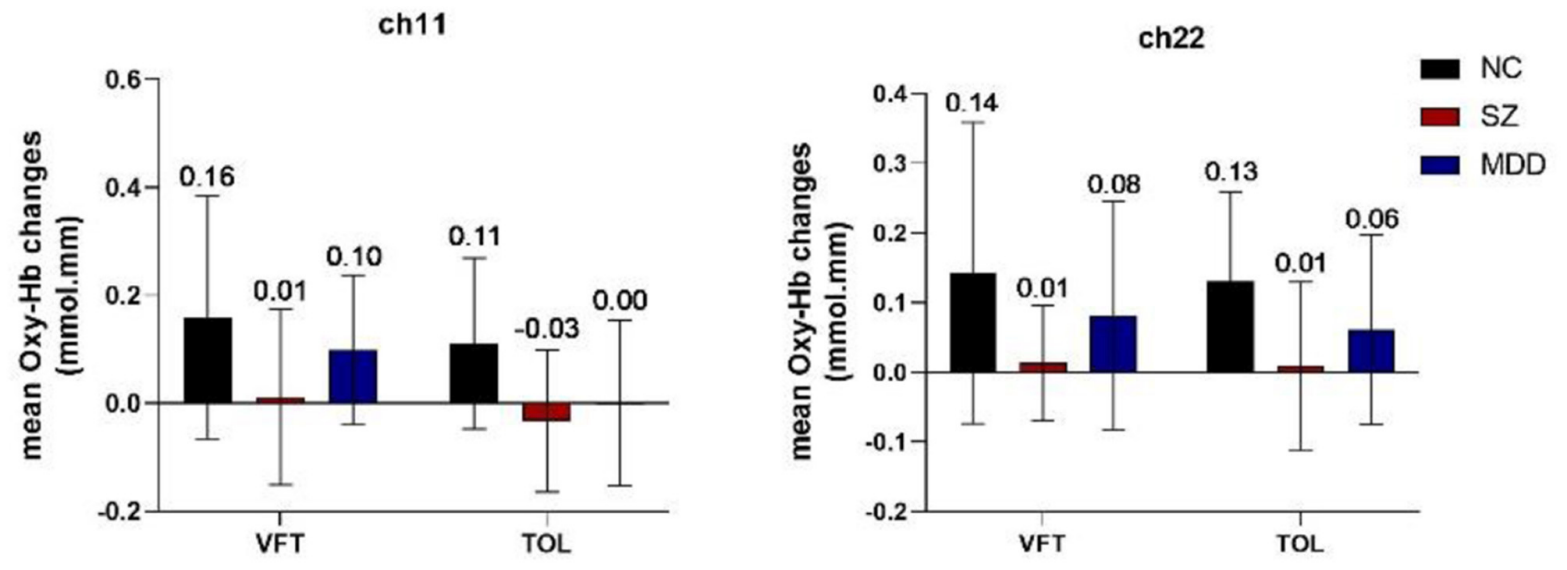
Mean [oxy-Hb] changes in ch11 and ch22.

**Figure 3:**
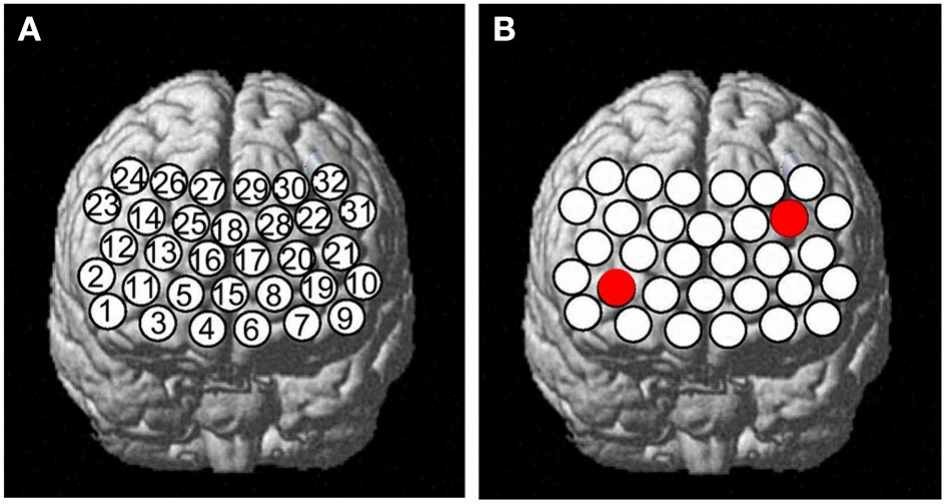
A panel **(A)** Thirty-two channels' positions and **(B)** shows ch11 and ch22.

**Figure 4:**
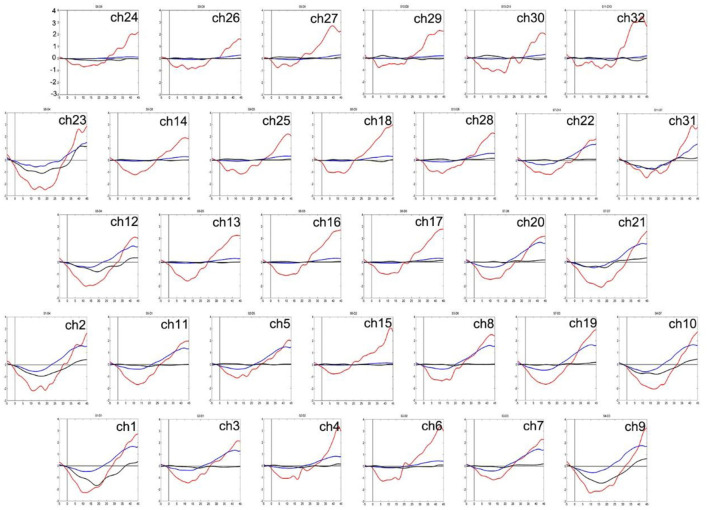
The time course of mean hemodynamic changes (*Z*-values) of 32 channels during the VFT task. The ordinate is the mean hemodynamic changes (*Z*-value, *10^−1^ mmol.mm), and the abscissa is the time course of the task. HCs (red); MDD patients (blue); SZ patients (black).

**Figure 5:**
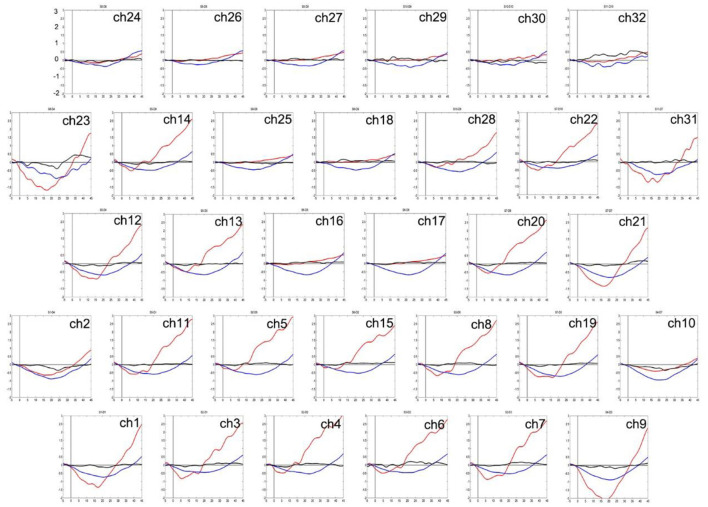
The time course of mean hemodynamic changes (*Z*-values) of 32 channels during the TOL task. The ordinate is the mean hemodynamic changes (*Z*-value, *10^−1^mmol.mm), and the abscissa is the time course of the task. HCs (red); MDD patients (blue); SZ patients (black).

Two-way ANOVA indicated a main effect of group in all channels, and a main effect of task in most channels. There was no significant interaction between group and task. *Post-hoc* comparisons showed that compared to HCs, both of the patient groups exhibited significantly decreased mean [oxy-Hb] changes in all channels. Especially in ch11 (*F* = 11.818, FDR *p* = 0.047) and ch22 (*F* = 10.830, FDR *p* = 0.027) in the DLPFC, SZ patients exhibited significantly decreased mean [oxy-Hb] changes than the MDD patients. In most channels, the mean [oxy-Hb] changes were higher for the VFT task than for the TOL task (see Figure 2). The positions of ch11 and ch22 are shown in Figure 3. Figures 4, 5 show the time course of mean hemodynamic changes (*Z*-values) of all channels during the VFT and TOL tasks, respectively.

## Discussion

To the best of our knowledge, this is the first fNIRS study to compare the different characteristics of brain activation between SZ and MDD patients during the VFT and TOL task. We found that SZ patients showed poorer performance on VFT than MDD patients and HCs, and the two patient groups showed poorer performance on TOL than HCs. fNIRS results indicated that the activation of the extensive PFC in the two patient groups was significantly lower than that in HCs during the two tasks. Particularly in certain channels in the DLPFC, SZ patients exhibited significantly decreased hemodynamic changes than MDD patients.

### VFT Performance and TOL Performance

In the VFT, the mean number of the correct items generated was significantly lower in SZ patients than in MDD patients and HCs. These results were consistent with previous reports (20, 24). The VFT, which emphasizes information processing and memorizing ability, required participants to do some relevant memory retrieval with a limited range. The results indicated that SZ patients have cognitive impairment in memory retrieval and attention. One study was inconsistent with our results, which showed no significant difference in VFT performance among the three groups (25). The possible reason is that the study used a simpler letter fluency version that had a high successful execution rate for subjects, including psychiatric patients. In our study, we used semantic a word fluency task that could distinguish patients with more severe cognitive impairment.

In the TOL task, the accuracy and the average response time, respectively, reflect the accuracy and efficiency of planning and problem-solving ability. The results showed that SZ patients performed poorly, both accuracy and efficiency were lower, which were similar to a previous study (26). However, there have been no previous fNIRS study of TOLs in MDD patients. Our results showed that MDD patients planned and dealt with problems less effectively than HCs but had higher accuracy than SZ patients, which suggested that executive function impairment was more severe in SZ patients and were consistent with a previous study (15). Perhaps the TOL task could better distinguish between mental disorders and HCs due to both patient groups showing poorer TOL performance.

Considering the impact of IQ on the results, a previous study suggested that despite the controls' higher IQ scores, the groups showed comparable performance in most parameters of the three cognitive tasks (27). Our participants had normal IQs and no statistical difference in years of education, so the effect of IQ on the results was not taken into account. The duration of illness may also affect the results. A 1.5-year longitudinal study found that cognitive function, daily living skills, social function, and social activity were nominally improved over a 1.5-year follow-up period in SZ patients (28). The patients involved in the present study had an average illness duration of more than 24 months, and they all had cognitive impairment. Further longitudinal research is needed to explore the relationship between cognitive dysfunction and duration of illness.

### Reduced PFC Activation in the Patient Groups During Cognitive Tasks

The present result indicated that PFC activation was more extensive during the VFT than during the TOL task. The HCs showed activation in PFC during the TOL task, but there was no significant activation in patient groups. This is an interesting result, and not in line with our expectations. This could be due to the difficulty of the TOL task, or the fact that other deep brain regions were activated during the TOL task but fNIRS could not detect it. Our research focused on the PFC, and some MRI studies found that the TOL task also activated the frontostriatal and the parietal cortex (29, 30). Our results suggested that the patients failed to recruit enough PFC resources with the task and did not show the expected activation of task-related areas exhibited by the HCs, which was consistent with a previous study (14).

Most previous fNIRS studies of the VFT showed reduced PFC activation in SZ patients and MDD patients compared with HCs (11, 31). However, it is difficult to distinguish between MDD and SZ using only the mean [oxy-Hb] change in fNIRS signal. Simultaneously performing two cognitive tasks, SZ patients exhibited significantly decreased hemodynamic changes than the MDD patients in certain channels in the DLPFC (ch11: *p* = 0.047; ch22: *p* = 0.027). The result indicated that the DLPFC is crucial for executive function. This was consistent with several fMRI studies that show that the PFC is reliably activated during planning tasks (32, 33).

Combining our results with some previous studies has found that SZ patients have poorer task performance and cognitive function than MDD patients, such as in the VFT (20, 24), the TOL task (15), and the n-back task (34), which reflect executive function and working memory. Our results showed that SZ patients exhibited significantly decreased hemodynamic changes than the MDD patients in specific regions in the DLPFC during the two tasks, which indicated that the DLPFC is crucial for executive function. A previous MRI study using the TOL task in MDD showed that visuospatial planning *per se* was associated with increased frontostriatal activity and visuospatial planning load was associated with increased parietal activity (29). Another MRI study in SZ found that the TOL task activated several brain regions, including the DLPFC, the inferior frontal gyrus, and the parietal cortex (30). In addition to the PFC, the differences between the two diseases in other areas of the brain such as the parietal lobe should also be explored.

## Limitations

This study has several limitations. First, the statistical significance notwithstanding, the sample size of the study was relatively small. Second, all of the patients enrolled were taking medications, and the effect of medications on the fNIRS data cannot be ruled out. Longitudinal follow-up research was also not performed. Third, fNIRS can only measure the distance between 3 and 5 cm below the scalp, and cannot detect deeper brain areas. Our study showed that combining the two cognitive tasks can distinguish between SZ and MDD patients in specific brain regions. It is possible that a combination of fNIRS and other techniques could better search for biomarkers to distinguish the two diseases.

## Conclusions

Patients with SZ and MDD have different levels of impairment in different cognitive domains and PFC activation was more extensive during the VFT than during the TOL task. SZ patients exhibited significantly decreased hemodynamic changes than the MDD patients in specific regions in the DLPFC, which could be used as targets for subsequent physical therapy such as rTMS. Further research is needed to determine the use of fNIRS for clinical evaluation and diagnosis.

## Data Availability Statement

The original contributions presented in the study are included in the article/supplementary material, further inquiries can be directed to the corresponding authors.

## Ethics Statement

The studies involving human participants were reviewed and approved by the Ethics Committees of Renmin Hospital of Wuhan University. The patients/participants provided their written informed consent to participate in this study.

## Author Contributions

WG, WH, LZ, and SC designed the study. XY and LY performed the study. XY undertook the imaging data analysis and wrote the first draft of the manuscript. WG and WH revised the manuscript.

## Funding

The study was supported by the Medical Science Advancement Program of Wuhan University (TFLC2018001).

## Conflict of Interest

The authors declare that the research was conducted in the absence of any commercial or financial relationships that could be construed as a potential conflict of interest. The reviewer LF declared a shared affiliation with the authors to the handling editor at time of review.

## Publisher's Note

All claims expressed in this article are solely those of the authors and do not necessarily represent those of their affiliated organizations, or those of the publisher, the editors and the reviewers. Any product that may be evaluated in this article, or claim that may be made by its manufacturer, is not guaranteed or endorsed by the publisher.

## Abbreviations

fNIRS: Functional near-infrared spectroscopy
VFT: Verbal fluency task
SZ: Schizophrenia
MDD: Major depressive disorder
TOL: Tower of London
PFC: Prefrontal cortex
DLPFC: Dorsolateral prefrontal cortex
VLPFC: Ventrolateral prefrontal cortex
FPPFC: Frontopolar prefrontal cortex
ch: Channel
HCs: Healthy controls
PANSS: Positive and Negative Syndrome Scale
HAMD: Hamilton Rating Scale for Depression
HAMA: Hamilton Rating Scale for Anxiety
Oxy-Hb: Oxygenated hemoglobin
deoxy-Hb: Deoxyhemoglobin
ANOVA: One-way analysis of variance
FDR: False discovery rate.
